# Trichlorido{μ-6,6′-dimeth­oxy-2,2′-[cyclo­hexane-1,2-diylbis(nitrilo­methanylyl­idene)]diphenolato}dimethano­l­copper(II)samarium(III)

**DOI:** 10.1107/S1600536812014523

**Published:** 2012-04-13

**Authors:** Yan Wang, Qian Zhang, Peng-Fei Yan, Guang-Feng Hou, Hong-Feng Li

**Affiliations:** a School of Chemistry and Materials Science, Heilongjiang University, Harbin 150080, People’s Republic of China

## Abstract

In the title hetero-dinuclear complex, [CuSm(C_22_H_24_N_2_O_4_)Cl_3_(CH_3_OH)_2_], the Cu^II^ cation is *N*,*N*′,*O*,*O*′-chelated by a 6,6′-dimeth­oxy-2,2′-[cyclo­hexane-1,2-diylbis(nitrilo­methanylyl­idene)]diphenolate ligand, and one Cl^−^ anion further coordinates to the Cu^II^ cation to complete the distorted square-pyramidal coordination geometry, while the Sm^III^ cation is chelated by four O atoms from the same ligand, and is further coordinated by two methanol mol­ecules and two Cl^−^ anions in an bicapped trigonal–prismatic geometry. Intra- and inter­molecular O—H⋯Cl hydrogen bonds are present in the structure.

## Related literature

For background to metallic Schiff base complexes and similar structures, see: Liu *et al.* (1990 ▶); Xu *et al.* (2011 ▶). For the synthesis of the ligand, see: Bao *et al.* (2010 ▶).
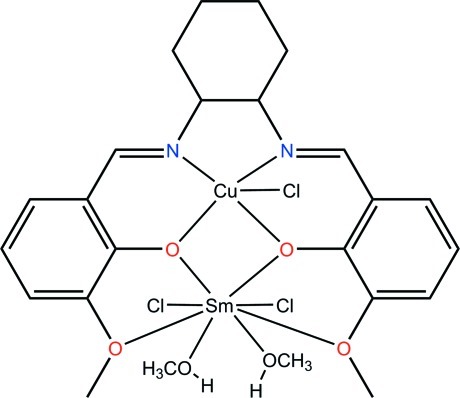


## Experimental

### 

#### Crystal data

[CuSm(C_22_H_24_N_2_O_4_)Cl_3_(CH_4_O)_2_]
*M* *_r_* = 764.77
Monoclinic, 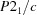

*a* = 7.5130 (15) Å
*b* = 26.712 (5) Å
*c* = 14.970 (5) Å
β = 110.21 (3)°
*V* = 2819.3 (12) Å^3^
*Z* = 4
Mo *K*α radiation
μ = 3.14 mm^−1^
*T* = 293 K
0.24 × 0.22 × 0.16 mm

#### Data collection

Rigaku R-AXIS RAPID diffractometer
Absorption correction: multi-scan (*ABSCOR*; Higashi, 1995 ▶) *T*_min_ = 0.475, *T*_max_ = 0.605
26818 measured reflections
6432 independent reflections
5702 reflections with *I* > 2σ(*I*)
*R*_int_ = 0.039

#### Refinement

*R*[*F* ^2^ > 2σ(*F* ^2^)] = 0.038
*wR*(*F* ^2^) = 0.096
*S* = 1.11
6432 reflections
340 parameters
H-atom parameters constrained
Δρ_max_ = 1.38 e Å^−3^
Δρ_min_ = −1.22 e Å^−3^

### 

Data collection: *RAPID-AUTO* (Rigaku, 1998 ▶); cell refinement: *RAPID-AUTO*; data reduction: *CrystalClear* (Rigaku/MSC, 2002 ▶); program(s) used to solve structure: *SHELXTL* (Sheldrick, 2008 ▶); program(s) used to refine structure: *SHELXTL*; molecular graphics: *SHELXTL*; software used to prepare material for publication: *SHELXTL*.

## Supplementary Material

Crystal structure: contains datablock(s) I, global. DOI: 10.1107/S1600536812014523/xu5492sup1.cif

Structure factors: contains datablock(s) I. DOI: 10.1107/S1600536812014523/xu5492Isup2.hkl

Additional supplementary materials:  crystallographic information; 3D view; checkCIF report

## Figures and Tables

**Table 1:** Hydrogen-bond geometry (Å, °)

*D*—H⋯*A*	*D*—H	H⋯*A*	*D*⋯*A*	*D*—H⋯*A*
O5—H5*A*⋯Cl3	0.83	2.20	3.016 (4)	169
O6—H6⋯Cl2^i^	0.82	2.45	3.251 (4)	166

Symmetry code: (i) 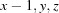.

**Fig. 1.**
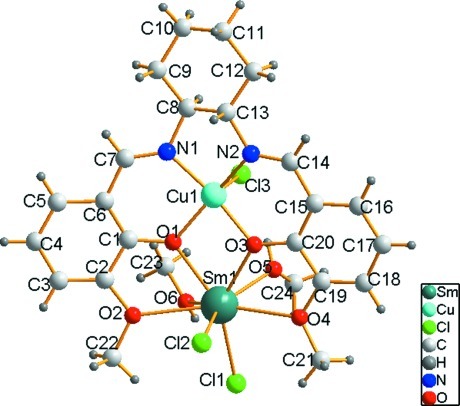
The crystal structure of the title compound.

## supplementary crystallographic information

### Comment

Metallic Schiff base complexes are of considerable interest because of the variable structures and potential application in magnetic, luminescence and electrochemical behavior (Liu *et al.*, 1990). We present here the sythesis and crystal structure of the title complex.

In the title complex, the Cu(II) ions is five-coordinated by two imino nitrogen atoms and two phenolate oxygen atoms from the ligand, and one chlorine atom. The Cu—N bond distances are 1.926 (4) and 1.931 (4) Å, and the Cu—O bond distances are in the range of 1.926 (3)—1.942 (3) Å, which in accordance with the reported values. The Sm(III) ion is eight-coordinated by four oxygen atoms from the ligand, two oxygen atoms of two methanol molecules and two Cl atoms. The Sm—O bond distances are in the range of 2.372 (3)—2.615 (3) Å, the Sm—Cl bond distances are 2.6637 (13) and 2.6940 (15) Å. (Fig.1, Table 1). The Sm—Cu distance is 3.4292 (9) Å. The positive charge of the Sm(III) and Cu(II) ions are balanced by the ligand *L*^2-^ and three chlorine anions (*L* = *N*,*N*'-bis(2-oxy-3-methoxybenzylidene) -1,2-diaminocyclohexane).

### Experimental

The salen-type ligand was synthesized following the reference (Bao *et al.* 2010). To a 1:1 MeOH/CH_2_Cl_2_ solution (20 mL) of LCuH_2_O (0.0980 g, 0.2 mmol) was added SmCl_3_˙6H_2_O (0.0728 g, 0.2 mmol) at the room temperature. After stirring for 12 h, the solution was filtered to remove the suspended particles. Yellow single crystals suitable for X-ray diffraction were obtained by slow diffusion of diethylether into the filtrate in five days. (CuClC_22_H_24_O_4_N_2_)SmCl_2_(CH_3_OH)_2_, Elemental Anal. Calc.: C, 37.69; H, 4.22; N, 3.66 wt%, Found: C, 37.68; H, 4.24; N, 3.64 wt%.

### Refinement

H atoms bound to C atoms were placed in calculated positions and treated as riding on their parent atoms, with C—H = 0.93–0.98 Å with *U*_iso_(H) = 1.2(1.5 for methyl)*U*eq(C). Hydroxy H atoms were located in a difference Fourier map and refined with the O—H bond distance restrained to 0.82 Å. The reflections (0 4 6), (2 9 4), (1 8 5), (3 6 3) and (-3 2 9) have been omitted during the refinement.

### Crystal data

**Table:**

[CuSm(C_22_H_24_N_2_O_4_)Cl_3_(CH_4_O)_2_]	*F*(000) = 1520
*M**_r_* = 764.77	*D*_x_ = 1.802 Mg m^−^^3^
Monoclinic, *P*2_1_/*c*	Mo *K*α radiation, λ = 0.71073 Å
Hall symbol: -P 2ybc	Cell parameters from 22347 reflections
*a* = 7.5130 (15) Å	θ = 3.0–27.5°
*b* = 26.712 (5) Å	µ = 3.14 mm^−^^1^
*c* = 14.970 (5) Å	*T* = 293 K
β = 110.21 (3)°	Block, yellow
*V* = 2819.3 (12) Å^3^	0.24 × 0.22 × 0.16 mm
*Z* = 4	

### Data collection

**Table:**

Rigaku R-AXIS RAPID diffractometer	6432 independent reflections
Radiation source: fine-focus sealed tube	5702 reflections with *I* > 2σ(*I*)
Graphite monochromator	*R*_int_ = 0.039
ω scan	θ_max_ = 27.5°, θ_min_ = 3.0°
Absorption correction: multi-scan (*ABSCOR*; Higashi, 1995)	*h* = −9→9
*T*_min_ = 0.475, *T*_max_ = 0.605	*k* = −34→34
26818 measured reflections	*l* = −19→19

### Refinement

**Table:**

Refinement on *F*^2^	Primary atom site location: structure-invariant direct methods
Least-squares matrix: full	Secondary atom site location: difference Fourier map
*R*[*F*^2^ > 2σ(*F*^2^)] = 0.038	Hydrogen site location: inferred from neighbouring sites
*wR*(*F*^2^) = 0.096	H-atom parameters constrained
*S* = 1.11	*w* = 1/[σ^2^(*F*_o_^2^) + (0.0215*P*)^2^ + 12.8071*P*] where *P* = (*F*_o_^2^ + 2*F*_c_^2^)/3
6432 reflections	(Δ/σ)_max_ = 0.007
340 parameters	Δρ_max_ = 1.38 e Å^−^^3^
0 restraints	Δρ_min_ = −1.22 e Å^−^^3^

### Special details

**Table:**

Geometry. All esds (except the esd in the dihedral angle between two l.s. planes) are estimated using the full covariance matrix. The cell esds are taken into account individually in the estimation of esds in distances, angles and torsion angles; correlations between esds in cell parameters are only used when they are defined by crystal symmetry. An approximate (isotropic) treatment of cell esds is used for estimating esds involving l.s. planes.
Refinement. omit 0 4 6 omit 2 9 4 omit 1 8 5 omit 3 6 3 omit -3 2 9 Refinement of F^2^ against ALL reflections. The weighted R-factor wR and goodness of fit S are based on F^2^, conventional R-factors R are based on F, with F set to zero for negative F^2^. The threshold expression of F^2^ > 2sigma(F^2^) is used only for calculating R-factors(gt) etc. and is not relevant to the choice of reflections for refinement. R-factors based on F^2^ are statistically about twice as large as those based on F, and R- factors based on ALL data will be even larger.

### Fractional atomic coordinates and isotropic or equivalent isotropic displacement parameters (Å^2^)

**Table:**

	*x*	*y*	*z*	*U*_iso_*/*U*_eq_	
Sm1	0.71298 (3)	0.826090 (8)	0.734742 (16)	0.03035 (8)	
Cu1	0.87065 (9)	0.94753 (2)	0.75822 (4)	0.03624 (15)	
Cl1	0.5519 (2)	0.73817 (5)	0.74276 (10)	0.0479 (3)	
Cl2	1.0014 (2)	0.78339 (6)	0.69456 (11)	0.0561 (4)	
Cl3	0.5786 (2)	0.97014 (5)	0.81281 (11)	0.0511 (3)	
C1	0.6853 (7)	0.91187 (19)	0.5636 (3)	0.0370 (10)	
C2	0.6128 (7)	0.8715 (2)	0.5017 (3)	0.0378 (11)	
C3	0.5577 (8)	0.8772 (2)	0.4044 (4)	0.0500 (14)	
H3	0.5102	0.8502	0.3641	0.060*	
C4	0.5742 (10)	0.9239 (2)	0.3674 (4)	0.0569 (16)	
H4	0.5351	0.9282	0.3018	0.068*	
C5	0.6472 (10)	0.9636 (2)	0.4261 (4)	0.0545 (15)	
H5	0.6580	0.9946	0.4001	0.065*	
C6	0.7065 (9)	0.9580 (2)	0.5259 (4)	0.0454 (12)	
C7	0.7905 (11)	1.0016 (2)	0.5827 (4)	0.0603 (17)	
H7	0.7938	1.0311	0.5504	0.072*	
C8	0.9401 (16)	1.0476 (3)	0.7301 (5)	0.100 (3)	
H8	0.8389	1.0570	0.7542	0.120*	
C9	0.9615 (11)	1.0941 (2)	0.6791 (5)	0.0625 (17)	
H9A	0.8385	1.1038	0.6344	0.075*	
H9B	1.0440	1.0872	0.6430	0.075*	
C10	1.042 (2)	1.1367 (3)	0.7461 (6)	0.123 (5)	
H10A	1.0891	1.1616	0.7126	0.148*	
H10B	0.9398	1.1519	0.7619	0.148*	
C11	1.1897 (12)	1.1251 (2)	0.8319 (7)	0.088 (3)	
H11A	1.1984	1.1525	0.8759	0.105*	
H11B	1.3075	1.1248	0.8188	0.105*	
C12	1.1795 (9)	1.0763 (2)	0.8835 (4)	0.0501 (14)	
H12A	1.3065	1.0661	0.9225	0.060*	
H12B	1.1070	1.0818	0.9252	0.060*	
C13	1.0883 (12)	1.0358 (2)	0.8137 (5)	0.079 (3)	
H13	1.1897	1.0281	0.7887	0.095*	
C14	1.1627 (7)	0.97096 (18)	0.9350 (4)	0.0411 (11)	
H14	1.2479	0.9936	0.9746	0.049*	
C15	1.1560 (7)	0.92122 (18)	0.9727 (3)	0.0341 (10)	
C16	1.2785 (7)	0.91120 (19)	1.0659 (3)	0.0391 (11)	
H16	1.3543	0.9368	1.1014	0.047*	
C17	1.2890 (8)	0.8648 (2)	1.1055 (4)	0.0437 (12)	
H17	1.3715	0.8591	1.1672	0.052*	
C18	1.1760 (8)	0.82578 (19)	1.0537 (4)	0.0414 (11)	
H18	1.1825	0.7942	1.0807	0.050*	
C19	1.0557 (7)	0.83442 (17)	0.9627 (3)	0.0341 (10)	
C20	1.0430 (6)	0.88190 (16)	0.9195 (3)	0.0295 (9)	
C21	0.9741 (10)	0.7477 (2)	0.9362 (5)	0.0627 (18)	
H21A	1.1034	0.7393	0.9453	0.094*	
H21B	0.8908	0.7257	0.8895	0.094*	
H21C	0.9521	0.7441	0.9953	0.094*	
C22	0.5202 (11)	0.7845 (2)	0.4921 (5)	0.0673 (19)	
H22A	0.3928	0.7916	0.4513	0.101*	
H22B	0.5195	0.7568	0.5328	0.101*	
H22C	0.5960	0.7762	0.4540	0.101*	
C23	0.2473 (10)	0.8815 (3)	0.6207 (6)	0.076 (2)	
H23A	0.1999	0.8947	0.6677	0.115*	
H23B	0.1428	0.8732	0.5642	0.115*	
H23C	0.3257	0.9061	0.6056	0.115*	
C24	0.4502 (11)	0.8428 (3)	0.8882 (6)	0.071 (2)	
H24A	0.3326	0.8590	0.8535	0.106*	
H24B	0.4856	0.8508	0.9546	0.106*	
H24C	0.4354	0.8072	0.8798	0.106*	
N1	0.8603 (8)	1.00248 (16)	0.6733 (3)	0.0533 (13)	
N2	1.0622 (6)	0.98642 (15)	0.8522 (3)	0.0394 (10)	
O1	0.7289 (5)	0.90241 (12)	0.6569 (2)	0.0406 (8)	
O2	0.5985 (6)	0.82768 (13)	0.5490 (2)	0.0442 (9)	
O3	0.9297 (5)	0.88600 (11)	0.8294 (2)	0.0335 (7)	
O4	0.9380 (5)	0.79875 (12)	0.9037 (2)	0.0376 (8)	
O5	0.5922 (5)	0.85941 (13)	0.8540 (3)	0.0458 (9)	
H5A	0.5831	0.8903	0.8488	0.055*	
O6	0.3557 (6)	0.83790 (16)	0.6567 (3)	0.0561 (10)	
H6	0.2791	0.8194	0.6687	0.067*	

### Atomic displacement parameters (Å^2^)

**Table:**

	*U*^11^	*U*^22^	*U*^33^	*U*^12^	*U*^13^	*U*^23^
Sm1	0.03588 (13)	0.02655 (12)	0.02773 (12)	−0.00480 (9)	0.00986 (9)	−0.00285 (9)
Cu1	0.0475 (4)	0.0268 (3)	0.0287 (3)	−0.0079 (2)	0.0058 (2)	0.0009 (2)
Cl1	0.0521 (8)	0.0312 (6)	0.0544 (8)	−0.0119 (5)	0.0106 (6)	−0.0019 (5)
Cl2	0.0495 (8)	0.0647 (9)	0.0612 (9)	−0.0009 (7)	0.0281 (7)	−0.0145 (7)
Cl3	0.0563 (8)	0.0338 (6)	0.0653 (9)	0.0011 (6)	0.0237 (7)	−0.0028 (6)
C1	0.043 (3)	0.041 (3)	0.025 (2)	0.002 (2)	0.010 (2)	0.0041 (19)
C2	0.037 (3)	0.046 (3)	0.032 (2)	−0.001 (2)	0.013 (2)	−0.001 (2)
C3	0.051 (3)	0.060 (4)	0.033 (3)	−0.005 (3)	0.007 (2)	−0.008 (2)
C4	0.070 (4)	0.067 (4)	0.028 (3)	0.001 (3)	0.011 (3)	0.004 (3)
C5	0.075 (4)	0.055 (3)	0.030 (3)	0.005 (3)	0.014 (3)	0.013 (2)
C6	0.057 (3)	0.041 (3)	0.034 (3)	0.000 (3)	0.012 (2)	0.003 (2)
C7	0.098 (5)	0.043 (3)	0.040 (3)	−0.011 (3)	0.025 (3)	0.009 (2)
C8	0.176 (10)	0.051 (4)	0.047 (4)	−0.047 (5)	0.006 (5)	0.005 (3)
C9	0.091 (5)	0.035 (3)	0.062 (4)	−0.015 (3)	0.028 (4)	0.003 (3)
C10	0.228 (13)	0.049 (4)	0.071 (5)	−0.047 (6)	0.025 (7)	0.008 (4)
C11	0.078 (5)	0.034 (3)	0.123 (7)	−0.023 (3)	−0.002 (5)	0.013 (4)
C12	0.054 (3)	0.037 (3)	0.060 (4)	−0.013 (3)	0.021 (3)	−0.009 (2)
C13	0.090 (5)	0.032 (3)	0.077 (5)	−0.026 (3)	−0.020 (4)	0.011 (3)
C14	0.041 (3)	0.032 (2)	0.043 (3)	−0.004 (2)	0.005 (2)	−0.008 (2)
C15	0.035 (2)	0.034 (2)	0.031 (2)	−0.0027 (19)	0.0079 (19)	−0.0062 (19)
C16	0.035 (3)	0.041 (3)	0.034 (2)	−0.001 (2)	0.002 (2)	−0.007 (2)
C17	0.044 (3)	0.048 (3)	0.030 (2)	0.002 (2)	0.001 (2)	0.002 (2)
C18	0.047 (3)	0.036 (3)	0.036 (3)	0.003 (2)	0.007 (2)	0.006 (2)
C19	0.035 (2)	0.031 (2)	0.036 (2)	−0.0002 (19)	0.012 (2)	−0.0045 (19)
C20	0.030 (2)	0.027 (2)	0.029 (2)	0.0007 (18)	0.0083 (18)	−0.0024 (17)
C21	0.063 (4)	0.029 (3)	0.072 (4)	0.001 (3)	−0.006 (3)	0.003 (3)
C22	0.086 (5)	0.059 (4)	0.053 (4)	−0.035 (4)	0.021 (3)	−0.026 (3)
C23	0.057 (4)	0.077 (5)	0.089 (5)	0.015 (4)	0.016 (4)	0.031 (4)
C24	0.093 (5)	0.060 (4)	0.086 (5)	−0.011 (4)	0.066 (5)	−0.011 (4)
N1	0.086 (4)	0.032 (2)	0.035 (2)	−0.013 (2)	0.012 (2)	0.0020 (18)
N2	0.039 (2)	0.0268 (19)	0.044 (2)	−0.0078 (17)	0.0038 (19)	−0.0006 (17)
O1	0.060 (2)	0.0315 (17)	0.0270 (16)	−0.0119 (16)	0.0108 (15)	−0.0015 (13)
O2	0.064 (2)	0.0354 (18)	0.0251 (16)	−0.0128 (17)	0.0055 (16)	−0.0079 (14)
O3	0.0392 (18)	0.0292 (16)	0.0270 (15)	−0.0041 (14)	0.0050 (13)	0.0010 (12)
O4	0.0423 (19)	0.0318 (17)	0.0328 (17)	−0.0052 (15)	0.0058 (15)	−0.0013 (13)
O5	0.053 (2)	0.0339 (18)	0.061 (2)	−0.0036 (16)	0.033 (2)	−0.0056 (17)
O6	0.044 (2)	0.057 (2)	0.064 (3)	−0.0005 (19)	0.015 (2)	0.013 (2)

### Geometric parameters (Å, º)

**Table:**

Sm1—O1	2.372 (3)	C11—H11A	0.9700
Sm1—O3	2.372 (3)	C11—H11B	0.9700
Sm1—O5	2.434 (4)	C12—C13	1.497 (8)
Sm1—O6	2.550 (4)	C12—H12A	0.9700
Sm1—O2	2.611 (3)	C12—H12B	0.9700
Sm1—O4	2.615 (3)	C13—N2	1.478 (7)
Sm1—Cl1	2.6637 (13)	C13—H13	0.9800
Sm1—Cl2	2.6940 (15)	C14—N2	1.277 (7)
Sm1—Cu1	3.4296 (9)	C14—C15	1.451 (7)
Cu1—O3	1.926 (3)	C14—H14	0.9300
Cu1—N1	1.926 (4)	C15—C16	1.407 (7)
Cu1—N2	1.931 (4)	C15—C20	1.411 (6)
Cu1—O1	1.942 (3)	C16—C17	1.365 (7)
Cu1—Cl3	2.6621 (17)	C16—H16	0.9300
C1—O1	1.343 (5)	C17—C18	1.398 (7)
C1—C6	1.388 (7)	C17—H17	0.9300
C1—C2	1.404 (7)	C18—C19	1.369 (7)
C2—C3	1.379 (7)	C18—H18	0.9300
C2—O2	1.390 (6)	C19—O4	1.389 (6)
C3—C4	1.388 (9)	C19—C20	1.412 (6)
C3—H3	0.9300	C20—O3	1.327 (5)
C4—C5	1.366 (9)	C21—O4	1.441 (6)
C4—H4	0.9300	C21—H21A	0.9600
C5—C6	1.412 (7)	C21—H21B	0.9600
C5—H5	0.9300	C21—H21C	0.9600
C6—C7	1.450 (8)	C22—O2	1.434 (6)
C7—N1	1.275 (7)	C22—H22A	0.9600
C7—H7	0.9300	C22—H22B	0.9600
C8—C13	1.394 (10)	C22—H22C	0.9600
C8—N1	1.477 (8)	C23—O6	1.416 (8)
C8—C9	1.497 (8)	C23—H23A	0.9600
C8—H8	0.9800	C23—H23B	0.9600
C9—C10	1.499 (10)	C23—H23C	0.9600
C9—H9A	0.9700	C24—O5	1.405 (7)
C9—H9B	0.9700	C24—H24A	0.9600
C10—C11	1.410 (12)	C24—H24B	0.9600
C10—H10A	0.9700	C24—H24C	0.9600
C10—H10B	0.9700	O5—H5A	0.8297
C11—C12	1.531 (9)	O6—H6	0.8237
			
O1—Sm1—O3	64.73 (11)	H10A—C10—H10B	107.3
O1—Sm1—O5	98.12 (12)	C10—C11—C12	119.0 (6)
O3—Sm1—O5	70.60 (12)	C10—C11—H11A	107.6
O1—Sm1—O6	83.82 (13)	C12—C11—H11A	107.6
O3—Sm1—O6	125.95 (13)	C10—C11—H11B	107.6
O5—Sm1—O6	71.99 (14)	C12—C11—H11B	107.6
O1—Sm1—O2	61.10 (11)	H11A—C11—H11B	107.0
O3—Sm1—O2	122.06 (11)	C13—C12—C11	110.8 (6)
O5—Sm1—O2	134.91 (13)	C13—C12—H12A	109.5
O6—Sm1—O2	66.59 (14)	C11—C12—H12A	109.5
O1—Sm1—O4	126.16 (11)	C13—C12—H12B	109.5
O3—Sm1—O4	61.92 (10)	C11—C12—H12B	109.5
O5—Sm1—O4	70.89 (13)	H12A—C12—H12B	108.1
O6—Sm1—O4	134.78 (13)	C8—C13—N2	112.3 (5)
O2—Sm1—O4	154.12 (12)	C8—C13—C12	119.6 (6)
O1—Sm1—Cl1	151.74 (9)	N2—C13—C12	117.6 (6)
O3—Sm1—Cl1	142.81 (8)	C8—C13—H13	100.9
O5—Sm1—Cl1	89.94 (9)	N2—C13—H13	100.9
O6—Sm1—Cl1	72.96 (10)	C12—C13—H13	100.9
O2—Sm1—Cl1	94.29 (8)	N2—C14—C15	125.8 (5)
O4—Sm1—Cl1	82.08 (8)	N2—C14—H14	117.1
O1—Sm1—Cl2	94.19 (10)	C15—C14—H14	117.1
O3—Sm1—Cl2	88.85 (9)	C16—C15—C20	118.6 (4)
O5—Sm1—Cl2	148.33 (10)	C16—C15—C14	117.5 (4)
O6—Sm1—Cl2	138.63 (11)	C20—C15—C14	123.9 (4)
O2—Sm1—Cl2	76.33 (10)	C17—C16—C15	121.6 (5)
O4—Sm1—Cl2	78.27 (9)	C17—C16—H16	119.2
Cl1—Sm1—Cl2	92.86 (5)	C15—C16—H16	119.2
O1—Sm1—Cu1	33.19 (8)	C16—C17—C18	120.2 (5)
O3—Sm1—Cu1	32.77 (7)	C16—C17—H17	119.9
O5—Sm1—Cu1	77.41 (9)	C18—C17—H17	119.9
O6—Sm1—Cu1	101.63 (10)	C19—C18—C17	119.5 (5)
O2—Sm1—Cu1	93.94 (8)	C19—C18—H18	120.2
O4—Sm1—Cu1	94.59 (7)	C17—C18—H18	120.2
Cl1—Sm1—Cu1	167.30 (4)	C18—C19—O4	124.9 (4)
Cl2—Sm1—Cu1	98.47 (4)	C18—C19—C20	121.6 (4)
O3—Cu1—N1	164.8 (2)	O4—C19—C20	113.4 (4)
O3—Cu1—N2	94.86 (16)	O3—C20—C15	124.1 (4)
N1—Cu1—N2	85.46 (19)	O3—C20—C19	117.3 (4)
O3—Cu1—O1	82.07 (14)	C15—C20—C19	118.5 (4)
N1—Cu1—O1	94.08 (17)	O4—C21—H21A	109.5
N2—Cu1—O1	166.45 (19)	O4—C21—H21B	109.5
O3—Cu1—Cl3	94.62 (11)	H21A—C21—H21B	109.5
N1—Cu1—Cl3	100.37 (18)	O4—C21—H21C	109.5
N2—Cu1—Cl3	98.07 (15)	H21A—C21—H21C	109.5
O1—Cu1—Cl3	95.33 (12)	H21B—C21—H21C	109.5
O3—Cu1—Sm1	41.82 (9)	O2—C22—H22A	109.5
N1—Cu1—Sm1	136.02 (14)	O2—C22—H22B	109.5
N2—Cu1—Sm1	136.67 (12)	H22A—C22—H22B	109.5
O1—Cu1—Sm1	41.94 (10)	O2—C22—H22C	109.5
Cl3—Cu1—Sm1	87.36 (3)	H22A—C22—H22C	109.5
O1—C1—C6	124.6 (5)	H22B—C22—H22C	109.5
O1—C1—C2	116.2 (4)	O6—C23—H23A	109.5
C6—C1—C2	119.2 (4)	O6—C23—H23B	109.5
C3—C2—O2	125.8 (5)	H23A—C23—H23B	109.5
C3—C2—C1	121.2 (5)	O6—C23—H23C	109.5
O2—C2—C1	113.0 (4)	H23A—C23—H23C	109.5
C2—C3—C4	119.1 (5)	H23B—C23—H23C	109.5
C2—C3—H3	120.4	O5—C24—H24A	109.5
C4—C3—H3	120.4	O5—C24—H24B	109.5
C5—C4—C3	120.8 (5)	H24A—C24—H24B	109.5
C5—C4—H4	119.6	O5—C24—H24C	109.5
C3—C4—H4	119.6	H24A—C24—H24C	109.5
C4—C5—C6	120.6 (5)	H24B—C24—H24C	109.5
C4—C5—H5	119.7	C7—N1—C8	124.3 (5)
C6—C5—H5	119.7	C7—N1—Cu1	126.6 (4)
C1—C6—C5	119.0 (5)	C8—N1—Cu1	109.0 (4)
C1—C6—C7	124.2 (5)	C14—N2—C13	123.6 (5)
C5—C6—C7	116.7 (5)	C14—N2—Cu1	125.1 (3)
N1—C7—C6	125.0 (5)	C13—N2—Cu1	111.2 (3)
N1—C7—H7	117.5	C1—O1—Cu1	124.5 (3)
C6—C7—H7	117.5	C1—O1—Sm1	129.6 (3)
C13—C8—N1	111.9 (6)	Cu1—O1—Sm1	104.87 (14)
C13—C8—C9	116.9 (7)	C2—O2—C22	117.5 (4)
N1—C8—C9	118.5 (6)	C2—O2—Sm1	120.1 (3)
C13—C8—H8	102.0	C22—O2—Sm1	122.5 (3)
N1—C8—H8	102.0	C20—O3—Cu1	125.3 (3)
C9—C8—H8	102.0	C20—O3—Sm1	128.2 (3)
C8—C9—C10	112.2 (6)	Cu1—O3—Sm1	105.41 (13)
C8—C9—H9A	109.2	C19—O4—C21	115.5 (4)
C10—C9—H9A	109.2	C19—O4—Sm1	118.7 (3)
C8—C9—H9B	109.2	C21—O4—Sm1	125.0 (3)
C10—C9—H9B	109.2	C24—O5—Sm1	132.4 (4)
H9A—C9—H9B	107.9	C24—O5—H5A	107.4
C11—C10—C9	117.0 (8)	Sm1—O5—H5A	109.5
C11—C10—H10A	108.1	C23—O6—Sm1	130.7 (4)
C9—C10—H10A	108.1	C23—O6—H6	102.8
C11—C10—H10B	108.1	Sm1—O6—H6	122.0
C9—C10—H10B	108.1		

### Hydrogen-bond geometry (Å, º)

**Table:**

*D*—H···*A*	*D*—H	H···*A*	*D*···*A*	*D*—H···*A*
O5—H5*A*···Cl3	0.83	2.20	3.016 (4)	169
O6—H6···Cl2^i^	0.82	2.45	3.251 (4)	166

Symmetry code: (i) *x*−1, *y*, *z*.
